# Timing of Tracheotomy in Mechanically Ventilated Critically Ill Morbidly Obese Patients

**DOI:** 10.1155/2014/840638

**Published:** 2014-09-15

**Authors:** Ahmad Alhajhusain, Ailia W. Ali, Asif Najmuddin, Kashif Hussain, Masooma Aqeel, Ali A. El-Solh

**Affiliations:** ^1^Department of Medicine, Division of Pulmonary, Critical Care, and Sleep Medicine, West Virginia University School of Medicine, Morgantown, WV, USA; ^2^Department of Medicine, Division of Pulmonary, Critical Care, and Sleep Medicine, University at Buffalo School of Medicine and Biomedical Sciences and the Veterans Affairs Medical Center, Buffalo, NY, USA; ^3^Medical Research, VA Western New York Healthcare System, Building 20 (151) VISN02, 3495 Bailey Avenue, Buffalo, NY 14215-1199, USA

## Abstract

*Background.* The optimal timing of tracheotomy and its impact on weaning from mechanical ventilation in critically ill morbidly obese patients remain controversial. *Methods.* We conducted a retrospective chart review of morbidly obese subjects (BMI ≥ 40 kg/m^2^ or BMI ≥ 35 kg/m^2^ and one or more comorbid conditions) who underwent a tracheotomy between July 2008 and June 2013 at a medical intensive care unit (ICU). Clinical characteristics, rates of nosocomial pneumonia (NP), weaning from mechanical ventilation (MV), and mortality rates were analyzed. *Results.* A total of 102 subjects (42 men and 60 women) were included; their mean age and BMI were 56.3 ± 15.1 years and 53.3 ± 13.6 kg/m^2^, respectively. There was no difference in the rate of NP between groups stratified by successful weaning from MV (*P* = 0.43). Mortality was significantly higher in those who failed to wean (*P* = 0.02). A cutoff value of 9 days for the time to tracheotomy provided the best balanced sensitivity (72%) and specificity (59.8%) for predicting NP onset. Rates of NP and total duration of MV were significantly higher in those who had tracheostomy ≥ 9 days (*P* = 0.004 and *P* = 0.002, resp.). *Conclusions.* The study suggests that tracheotomy in morbidly obese subjects performed within the first 9 days may reduce MV and decrease NP but may not affect hospital mortality.

## 1. Introduction

Tracheotomy is one of the most frequent procedures performed in critically ill patients. It has been advocated for those requiring prolonged mechanical ventilation because it facilitates weaning by decreasing the work of breathing in patients with limited reserve [1, 2], decreases the requirement for sedation [3], and may allow for earlier patient mobilization, feeding, and physical and occupational therapy. Recent studies have suggested that tracheotomy results in fewer oral-labial ulcerations, improves pulmonary toileting, and lowers incidence of pulmonary infections [4]. Furthermore, newer techniques such as percutaneous dilatation tracheotomy (PDT) have been shown to be cost-effective and safe, offering clinicians an effective alternative to surgical tracheostomy [5, 6]. Tracheotomy, however, is not devoid of risks. Complications may include hemorrhage, stoma infections, pneumothorax, subcutaneous emphysema, tracheal stenosis, tracheomalacia, and rarely death [7]. Hence, placement of tracheotomy should always take into consideration the benefit-risk tradeoff of the procedure.

As such, optimal timing for tracheotomy remains a subject of debate and continued investigation. Despite earlier studies suggesting benefits from early tracheotomy (within 2 to 10 days after intubation) [8, 9], a recent randomized trial did not find any mortality benefit from such a practice [10]. The current strategy is to implement an individualized approach taking into consideration the patient underlying comorbidities, reason for mechanical ventilation (MV), and potential complications of the procedure. One particular group that has consistently been associated with prolonged MV and ICU length of stay (LOS) is the morbidly obese patients [11, 12]. With a large and increasing population of obese mechanically ventilated patients, placement of tracheostomy represents a challenge because of the potentially higher complication rate with either surgical or percutaneous dilatory tracheotomy [13]. Despite the theoretical advantages of tracheostomy over translaryngeal intubation, few studies have been conducted in this population to determine the impact of early tracheostomy on patient outcomes. We hypothesize that early tracheostomy in morbidly obese patients requiring mechanical ventilation is associated with reduced duration of MV. The current study was conducted to determine the effect of early tracheostomy on clinical outcomes in ventilated morbidly obese patients.

## 2. Materials and Methods

### 2.1. Study Design

We conducted a retrospective chart review of all morbidly obese subjects (body mass index (BMI) ≥ 40 kg/m^2^ or BMI ≥ 35 kg/m^2^ and one or more comorbid conditions) admitted to the medical intensive care unit (ICU) between July 2008 and June 2013 who required tracheotomy. The ICU is a 25-bed closed medical unit staffed by board certified intensivists, a critical care fellow, and three senior residents. The decision to perform tracheotomy was made during medical rounds every morning. With respect to indications for tracheotomy, the following criteria or their combination were used: (1) subjects without any provision of liberation from MV, (2) prolonged MV, and (3) weaning failure.

Exclusion criteria included subjects <18 years of age, preexistent tracheotomy, admission for elective tracheotomy, or a do-not-resuscitate order. For those with more than one ICU admission, only the first event was included in the analysis to ensure independence of observation. Study approval was obtained from the local Institutional Review Board prior to initiation of the study. Written informed consent was waived due to the retrospective nature of the study.

### 2.2. Data Collection

Data collected included age, gender, BMI, cause and duration of MV, and APACHE II score on admission to the ICU. Medical records were analyzed also for nosocomial pneumonia (NP), ICU LOS, and hospital mortality. NP was defined according to the Centers for Disease Control and Prevention (CDC) criteria [14]. Successful weaning was defined as weaning from MV for more than 72 hours. The usual criteria for reinstating MV were the development of increased signs of respiratory work, inability to protect the airway, persistent low SaO2 < 90% with FiO2 > 50%, or severe arterial blood gas deterioration. Subjects who failed to wean following tracheotomy were transferred to long-term care facility if there were no other active clinical diseases.

### 2.3. Statistical Analysis

The primary outcome measure was duration of mechanical ventilation. Secondary outcomes included ICU and hospital length of stay, incidence of NP, and hospital mortality. Continuous variables were presented as mean ± standard deviation (SD) or median (interquartile range, IQR). Categorical variables were presented as number and percent. Continuous variables for the two groups were compared with Student's *t*-test for normally distributed data or the Mann-Whitney *U* test otherwise. Qualitative or categorical variables were compared with chi-square test or Fisher's exact test. Using NP as the classification variable, optimal time for tracheotomy was determined by the receiver operator characteristic (ROC) curve. The inflection point of the graph was chosen as the cutoff value because, at this threshold, there are an equal number of false-positive and false-negative results [15]. The optimal time cutoff value was defined using the Youden index [16]. Subjects who had a tracheotomy before the optimal time cutoff value formed the early tracheotomy group while those who had the procedure after were referred to as the late tracheotomy group. The number of deaths in each group was compared with the Fisher exact test and survival time with Kaplan-Meier curve and a log-rank test. A Cox regression analysis was conducted to identify risk factors for hospital mortality. Factors found to be significant in univariate analysis were included as independent variables. A multicollinearity test was performed using the variance inflation factor to assess the degree of correlation between covariates. A difference was considered statistically significant when the alpha probability was less than 0.05 (all two-tailed).

## 3. Results

**Figure 1:**
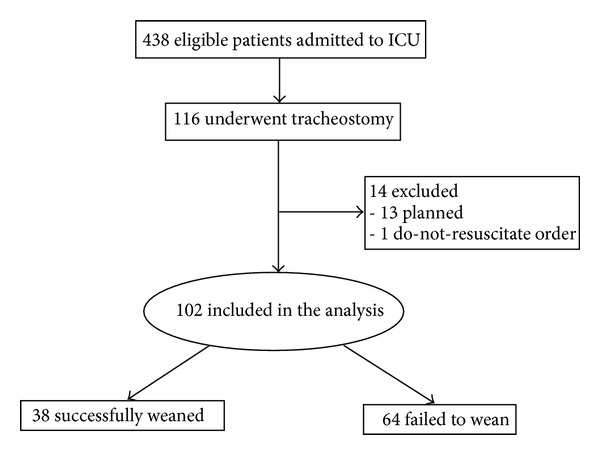
Study flow diagram.

**Figure 2:**
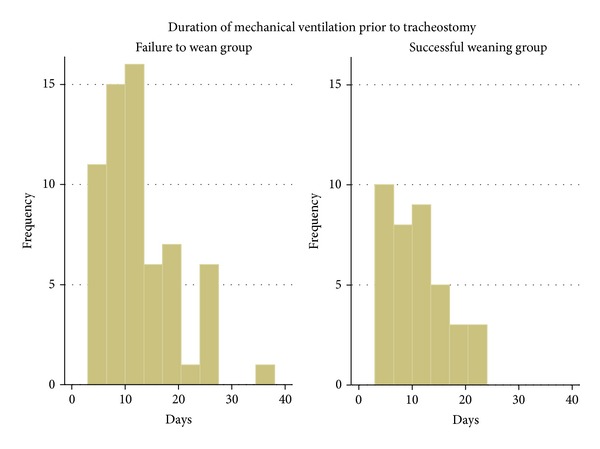
Histograms of time to tracheotomy in the successful weaning and failure to wean groups.

A total of 438 morbidly obese subjects were admitted to the ICU during the study period. Of 116 subjects who underwent tracheotomy, 14 subjects were excluded (13 for planned tracheotomy and one for DNR status) leaving 102 for statistical analysis (Figure 1). During the course of hospitalization, 38 (37%) were successfully weaned from mechanical ventilation and 64 (63%) failed to wean.  Table 1 displays the demographic and clinical characteristics of both groups. No significant difference in gender, BMI, APACHE II scores, or the type of tracheotomy was observed between the successful weaning and failure to wean groups. However subjects who failed to wean were significantly older and had a higher burden of comorbidities than those who were liberated successfully from MV (*P* = 0.007 and *P* = 0.003, resp.).  Figure 2 shows the distributions of the time to tracheotomy for both groups. The average time for those who weaned from MV was 10.9 ± 5.3 days compared to 12.3 ± 7.0 days for those who remained ventilator dependent (*P* = 0.29).

With respect to clinical outcome, the incidence of NP was comparable in both the failure to wean and the successful weaning groups (*P* = 0.82). However, the total duration of MV was significantly longer in the failure to wean group compared with the successful weaning group (*P* = 0.002). Similarly, the ICU LOS, hospital LOS, and hospital mortality were significantly higher in those who were failed to wean than those who were liberated from ventilatory support (Table 2).

**Figure 3:**
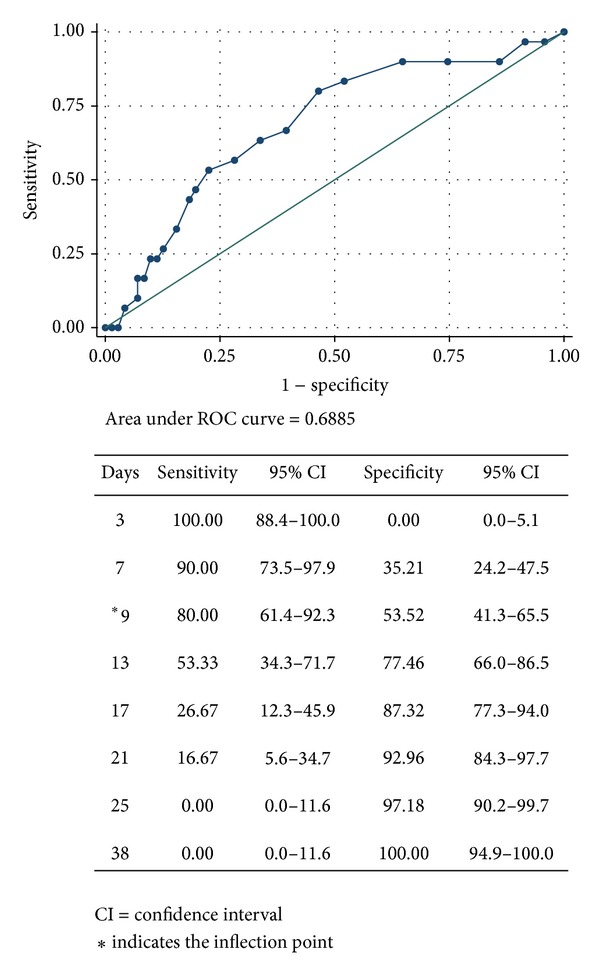
Receiver operator characteristic curve depicting optimal timing for tracheostomy.

**Figure 4:**
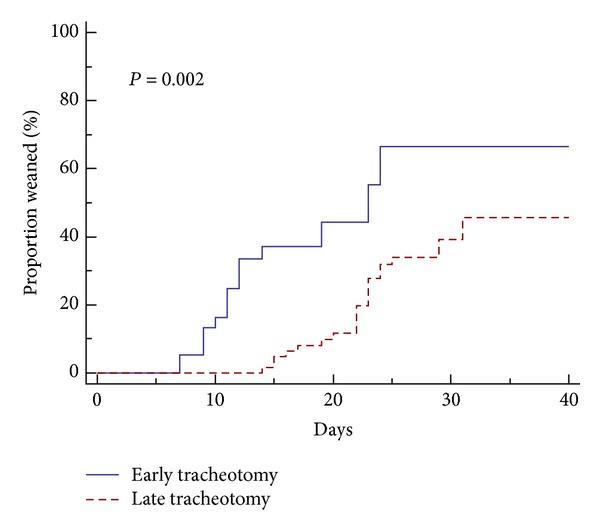
Kaplan-Meier curve of weaning time until successful liberation from mechanical ventilation.

The cutoff points of time to tracheotomy to predict NP event with their corresponding specificity and sensitivity are shown in Figure 3. Using 9 days as a cutoff for MV prior to tracheotomy, 39 (38%) subjects were categorized as having early tracheotomy and 63 (62%) as having late tracheotomy. The distribution of age, gender, underlying comorbidities, and severity of illness was comparable between the two groups (Table 3). Time to liberation from MV was significantly shorter in the early versus the late tracheotomy group (*P* = 0.002) (Figure 4). Subjects who underwent early tracheotomy received a mean of 15.1 ± 8.2 total days of respiratory support compared to 27.2 ± 10.9 days in those with late tracheotomy (*P* < 0.001). The total ICU LOS and hospital LOS were significantly longer in the late tracheotomy group than in the early tracheotomy group (*P* < 0.001). The incidence of NP was also significantly higher in the late tracheotomy group (*P* = 0.004). No significant difference in isolated microorganisms was found between the early and late tracheotomy groups. The analysis did not differ had we used the median duration of mechanical ventilation until tracheotomy (10 days) as a cutoff between early and late tracheotomy (Table 4) instead of the cutoff defined by the ROC.

Survival analysis showed no statistically significant difference in hospital mortality between the early tracheotomy and the late tracheotomy groups (*P* = 0.23). In multivariate analysis, hospital mortality was independently associated with weaning from MV (odds ratio (OR) 0.29; 95% confidence interval (CI) 0.09–0.91) and disease burden index (OR 1.31; 95% CI 1.06–1.62) (Table 5).

## 4. Discussion

Our data demonstrate that early tracheotomy was associated with reduced duration of MV, shorter ICU LOS, and a lower incidence of NP in critically ill morbidly obese patients.

The decision to institute tracheotomy in mechanically ventilated patients has been subject of a long debate between those who support early intervention citing the benefits of early liberation from MV and those who argue against this approach for lack of supportive evidence. Limitations in study designs and heterogeneity in patients' characteristics have hindered a consensus building toward a standardized frame for tracheotomy timing. In the case of morbidly obese critically ill patients, the decision is more challenging given the higher complication rate of this procedure in this population [17]. To date, no randomized trial of tracheotomy time has been completed in the morbidly obese patients. Two European multicenter randomized trials examining the role of early tracheotomy in clinical outcomes have not addressed this specific population or provided information on the BMI of their participants [10, 18]. Patient selection and predictive algorithm for prolonged MV become therefore paramount to the timing of tracheotomy. As a case in point, patients with severe neurological damage or traumatic brain injury require a distinct approach than other critically ill patients due to low frequency of early successful extubation [19, 20]. In these cases, early tracheotomy resulted in reduced mechanical ventilation time and decreased ICU LOS. Our observations are no different from these studies. Several clinical investigations have documented a prolonged duration of artificial ventilation in critically ill morbidly obese patients [11, 21, 22]. The increased dependency on MV in this population has been attributed to the increased work of breathing emanating from impaired respiratory mechanics, neuromuscular strength, and ventilatory drive [23]. In addition, morbidly obese patients experience high prevalence of sleep apnea which can lead to respiratory decompensation as a result of residual sedation, necessitating reintubation. The insertion of tracheotomy decreases pressure time product and reduces intrinsic positive end-expiratory pressure while improving ventilator synchrony [2, 24]. These changes, along with improved respiratory toileting and reduced requirement for sedation, may facilitate weaning.

Interestingly, there are inconsistencies concerning potential correlation between the timing of tracheotomy and pneumonia. In a case control study of 185 patients who underwent a surgical tracheotomy, the rate of ventilator associated pneumonia was significantly lower when tracheotomy was performed within 7 days after admission to the ICU [25]. Other retrospective studies found similar results [19, 26]. Consistent with our observations, the reduced rate of pneumonia may be attributed to the normal closure of vocal cords following removal of the endotracheal tube resulting in reduced risk of oropharyngeal aspiration. Moreover, early tracheotomy foreshortens length of MV and assists with early mobilization hence eliminating a major risk factor for NP. However, many prospective randomized trials failed to demonstrate an association between pneumonia and early tracheotomy [18, 27]. The most recent study by Terragni et al. [18] revealed no significant difference in the incidence of ventilator associated pneumonia (VAP) between an early and a late tracheotomy group. However, as outlined by Griffiths et al. [28], the heterogeneity of studied population and definitions of VAP and early tracheotomy limit some of the reproducibility of these results.

Whether early tracheotomy can decrease mortality remains unclear. In line with our results, a recent meta-analysis including seven randomized controlled trials studies found that the timing of tracheotomy was not correlated with hospital mortality [29]. Our study was not powered to examine mortality. However unsuccessful weaning was associated with a worse outcome in critically ill obese patients. A higher incidence of acute respiratory distress syndrome and acute kidney injury has been reported in this population [30]. In addition, prolonged ventilation usually entails more frequent invasive monitoring which predisposes the morbidly obese individuals to increased complications. A head-to-head comparison comparing extended MV in morbidly obese versus nonobese patients will be needed to settle this question.

In the current study, we did not report on the complications associated with tracheostomy in this population. However, we have previously observed a 25% complication rate related to tracheostomy in morbidly obese patients with an estimated mortality of 2% [17]. The most common complications were minor bleeding, stoma infection, and cuff leak. While the majority of these are considered nonlife threatening, loss of airway patency is particularly a catastrophic event in this group due to limited oxygen reserve.

Our current study has several limitations that need to be addressed. First, the design of the study introduces an inherent selection bias when comparing patients who are not randomized to selected treatment. Second, our data is generated from a single tertiary care center, which may limit the applicability of our findings to other critical care settings. Third, a physician-dependent bias due to different decision making for the timing of tracheotomy cannot be excluded. Fourth, most of the patients were discharged from the ICU without being decannulated. This may explain the relatively high hospital mortality in our study. It has been previously reported that lack of decannulation may lead to higher mortality when being compared to patients being decannulated before discharge [31]. Fifth, we have restricted our analysis to the time of hospitalization. Hence, weaning from ventilation may have occurred after hospital discharge.

In summary, the ever-expanding population of obese adults will result in an increased amount of surgical procedures being performed on these patients. In an effort to reduce morbidity, early tracheotomy may reduce total MV duration, ICU LOS, and incidence of pneumonia in critically ill morbidly obese patients. However, early tracheotomy may not reduce hospital mortality. These findings emphasize the need for an adequately supported multicenter trial to examine timing of this procedure in this special population.

## Figures and Tables

**Table 1:** Baseline characteristics of the study population.

	Successful weaning *N* = 38	Failure to wean *N* = 64	*P* value
Age, years	51.1 ± 13.2	59.4 ± 15.4	0.007
Gender (M/F)	15/23	27/37	0.83
BMI (kg/m^2^)	55.6 ± 14.2	52.1 ± 13.2	0.2
Charlson index	4 (3–5)	6 (4–8)	0.003
Comorbidities			
Chronic heart diseases, *n* (%)	6 (16)	17 (27)	0.23
Chronic pulmonary diseases, *n* (%)	6 (16)	22 (34)	0.07
Hypertension, *n* (%)	28 (74)	45 (70)	0.72
Diabetes mellitus, *n* (%)	20 (53)	29 (45)	0.54
Renal insufficiency, *n* (%)	7 (18)	20 (31)	0.16
Underlying causes for mechanical ventilation			
Cardiac failure, *n* (%)	4 (11)	9 (14)	0.83
Sepsis, *n* (%)	14 (37)	26 (41)	0.87
Respiratory failure, *n* (%)	15 (39)	20 (31)	0.52
Gastrointestinal∗, *n* (%)	2 (5)	2 (3)	0.99
Neurologic^†^, *n* (%)	3 (8)	7 (11)	0.87
Type of procedure			0.68
Surgical, *n* (%)	22 (58)	41 (64)	
Percutaneous, *n* (%)	16 (42)	23 (36)	
Timing to tracheotomy, days	10.9 ± 5.3	12.3 ± 7.0	0.29
PaO_2_/F_I_O_2_ at the time of tracheotomy	169.9 ± 97.9	180.1 ± 107.4	0.63
APACHE II	12.5 ± 5.9	14.2 ± 5.1	0.13

*Underlying gastrointestinal causes for mechanical ventilation included pancreatitis, diffuse colitis, and cholecystitis.

^†^Underlying neurologic causes for mechanical ventilation included cerebrovascular accidents and seizure disorders.

**Table 2:** Clinical outcomes stratified by weaning success.

	Successful weaning *N* = 38	Failure to wean *N* = 64	*P* value
Nosocomial pneumonia, *n* (%)	12	18	0.82
Total duration of mechanical ventilation, days	18.1 ± 6.9	25.2 ± 12.8	0.002
ICU length of stay, days	20.1 ± 7.6	24.9 ± 10.8	0.01
Hospital length of stay, days	31.7 ± 16.1	39.7 ± 16.7	0.02
Hospital mortality, *n* (%)	5 (13)	23 (36)	0.01

**Table 3:** Demographic and clinical characteristics of study population stratified by ROC-derived optimal time to tracheotomy.

	Early tracheotomy *N* = 39	Late tracheotomy *N* = 63	*P* value
Age, years	58.1 ± 14.9	55.2 ± 15.2	0.34
Gender (M/F)	15/24	27/36	0.68
BMI (kg/m^2^)	56.9 ± 15.4	51.2 ± 12.0	0.04
APACHE II	13.4 ± 5.6	13.7 ± 5.5	0.78
Charlson comorbidity index	5 (3–7)	5 (4–8)	0.85
Nosocomial pneumonia, *n* (%)	5 (13)	25 (39)	0.004
Total duration of mechanical ventilation, days	15.1 ± 8.2	27.2 ± 10.9	<0.001
ICU length of stay, days	16.6 ± 7.6	27.2 ± 9.1	<0.001
Hospital length of stay, days	27.6 ± 16.3	39.2 ± 15.4	<0.001
Hospital mortality, *n* (%)	11 (28)	17 (30)	0.89

**Table 4:** Demographic and clinical characteristics of study population stratified by median duration of mechanical ventilation until tracheotomy.

	Early tracheotomy *N* = 44	Late tracheotomy *N* = 58	*P* value
Age, years	57.6 ± 15.8	55.3 ± 14.6	0.45
Gender (M/F)	17/27	25/33	0.8
BMI (kg/m^2^)	55.8 ± 14.9	51.6 ± 12.4	0.13
APACHE II	13.5 ± 5.3	13.6 ± 5.7	0.95
Charlson comorbidity index	5 (3–7)	5 (4–8)	0.67
Nosocomial pneumonia, *n* (%)	6 (14)	24 (41)	0.002
Total duration of mechanical ventilation, days	15.9 ± 8.1	28.6 ± 11.2	<0.001
ICU length of stay, days	17.2 ± 7.7	27.6 ± 9.3	<0.001
Hospital length of stay, days	27.9 ± 15.8	39.8 ± 15.6	<0.001
Hospital mortality, *n* (%)	12 (27)	16 (28)	0.85

**Table 5:** Factors associated with hospital mortality.

	Odds ratio	*P* value	95% confidence interval
Age	1.00	0.173	0.93–1.01
APACHE II	1.03	0.203	0.85–1.09
Charlson index	1.31	0.013	1.06–1.62
Successful weaning	0.29	0.033	0.09–0.91
